# Thrombin generation’s role in predicting coronary disease severity

**DOI:** 10.1371/journal.pone.0237024

**Published:** 2020-08-07

**Authors:** Boaz Elad, Ofir Koren, Wasin slim, Yoav Turgeman, Gilat Avraham, Naama Schwartz, Mazen Elias

**Affiliations:** 1 Internal Medicine C, Emek Medical Center, Afula, Israel; 2 Heart Institute, Emek Medical Center, Afula, Israel; 3 Bruce Rappaport Faculty of Medicine, Technion-Israel Institute of Technology, Haifa, Israel; 4 Emergency Department, Emek Medical Center, Afula, Israel; 5 School of Public Health, University of Haifa, Haifa, Israel; Universite de Liege (B34), BELGIUM

## Abstract

**Background:**

Thrombin, a key enzyme of the clotting system, is involved in thrombus formation, platelet activation, and atherosclerosis, thereby possessing a central role in the pathogenesis of ischemic heart disease. Studies have shown an association between thrombin generation (TG) and cardiovascular morbidity and mortality, but results have been equivocal. Our aim was to study the predictive ability of TG assay in evaluating coronary stenosis severity.

**Methods:**

In this prospective study we recruited patients with acute coronary syndrome (ACS) or acute chest pain (without evidence of myocardial injury) planned for coronary angiography. Thrombin generation was evaluated by Calibrated Automated Thrombogram (CAT) prior to angiography. Primary end points were significant coronary stenosis and the Syntax I score evaluated by coronary angiography.

**Results:**

From April 2018 through September 2019, we recruited 128 patients. In the primary analysis there was no significant association between TG and significant coronary stenosis nor between TG and syntax I score, however, there was a positive correlation between peak height and troponin peak (Spearman correlation coefficient 0.194, P-value = 0.035). In sub-group analysis, the chest pain group bare no association between TG and coronary stenosis. In unstable angina group there was an association between peak height and significant coronary stenosis (P-value = 0.029), and in non ST-elevation myocardial infarction group, TG values possessed a relatively good predictive ability of significant coronary stenosis (area under the receiver operating characteristic curve of ~65%) and a positive correlation between both lag time and ttpeak with the syntax I score was noticed (Spearman correlation coefficient 0.31, P-value = 0.099 and Spearman correlation coefficient 0.37, P-value = 0.045 respectively).

**Conclusion:**

In patients with acute chest pain, TG values, evaluated by CAT, do not predict severity of coronary stenosis, nor do they possess prognostic value. Yet, in ACS patients, TG may have the ability to predict coronary disease severity.

## Background

Thrombin, a key enzyme in the clotting system, is involved in thrombus formation, platelet activation, and atherosclerosis. The enzyme seems to possess a central role in the formation of ischemic heart disease (IHD), and specifically, in acute coronary syndrome (ACS), usually caused by damaged atherosclerotic plaque which enables clotting factors and platelets to form a thrombus [1,2]. In order to evaluate patients’ thrombotic phenotype, evidence shows that thrombin generation (TG) tests may be more effective than "traditional" tests (i.e Prothrombin time, Partial Thromboplastin Time). Calibrated Automated Thrombogram (CAT), a fluorogenic TG assay, can be used to measure TG in multiple samples simultaneously and is an effective tool for evaluating TG [3,4]. Former work has found a correlation between TG tests, in vivo and ex-vivo, and cardiovascular morbidity and mortality, but the results have thus far been equivocal [1,2,5–9]. IHD is a disease with extensive rates of morbidity and mortality worldwide [10], therefore, it seems very compelling to further explore the correlation between TG, measured by a simple non-invasive test, and IHD. Evaluating IHD severity by coronary angiography is considered the gold standard technique. Yet, to our knowledge, correlation between TG assays and coronary stenosis, evaluated by angiography, has never been explored. Thus, our aim in this study was to evaluate the efficacy of TG tests in estimating coronary stenosis severity by angiography.

## Methods

### Study population

In this prospective, single-center study, we enrolled patients aged ≥18-years old, hospitalized with suspected ACS (unstable angina (UA), non-ST elevation myocardial infarction (NSTEMI), STEMI) or acute chest pain (without evidence of myocardial injury), and who were planned for coronary angiography. Patients were recruited from the emergency department, internal medicine wards and cardiology department of the Emek Medical Center, in Afula, Israel. ST elevation myocardial infarction (STEMI) patients did not participate. Exclusion criteria included patients with ACS in the former 6 months, known thrombophilia/coagulopathy, liver cirrhosis or decompensated liver disease, active oncological disease, use of anti-coagulation drugs, and pregnancy. Demographics, comorbidities and blood results were meticulously collected from electronics records. The study was approved by the hospital’s ethics committee and all patients signed an informed consent form. The chest pain group included patients without evidence of myocardial injury, defined by repeated normal troponin T levels and unfulfilled UA criteria [11].

### Thrombin generation and other laboratory tests

TG evaluation was performed prior to anticoagulation treatment and coronary angiography, with the use of the ex-vivo CAT assay (Thrombinoscope™ B.V., Maastricht, the Netherlands) for analysis of platelet-poor plasma (PPP). Six milliliters of blood were collected in two tubes containing sodium citrate. To obtain PPP, the samples were centrifuged twice, first for 15 minutes at 2500 revolutions per minute (RPM) and then for 10 minutes at 2000 RPM. The plasma was stored at −70°C for later analysis. According to the manufacturer's instructions, measurements were conducted in 80 μl of PPP triggered by 20 μl PPP-reagent (4 μmol phospholipid and 5 pmol tissue factor). Measurements were calibrated with 20 μl Thrombin Calibrator, Fluorogenic substrate (20 μl) were added to sample. The thrombogram generated four parameters: lag time (in minutes), time to peak (TTpeak) (in minutes), peak height (nM) and endogenous thrombin potential (ETP) (in nM*minute) representing the area under the TG curve [12,13]. Troponin T levels (pg/ml) were collected from electronical charts (before angiography). Troponin <14 was considered as 0.

### Coronary artery stenosis

Coronary stenosis was evaluated by coronary angiography. A coronary lesion was considered significant if stenosis was ≥ 70% or, in the case of the left main coronary artery, ≥ 50%. Syntax I score was calculated as well.

### Outcomes

Primary outcomes were significant coronary lesion and the syntax I score. Secondary outcomes were a composite of major adverse cardiovascular events (MACE) and Troponin T peak.

MACE definition: cardiovascular death, revascularization, acute coronary syndrome, ischemic cerebrovascular accident, heart failure hospitalization.

### Statistical analysis

Categorical variables are presented as frequency (%) and continuous variables as mean (SD) [median, interquartile range (IQR)]. Demographic differences between patients with and without significant coronary stenosis were tested using a Chi-square test or Fisher’s exact test for categorial variables. For continuous variables, the Student’s t-test or Mann Whitney U test were implemented. The associations between the TG parameters and the Syntax I score, and the maximum troponin value (separately), were examined using Spearman correlation.

The area under the receiver operating characteristic (ROC) curve (AUC) was estimated for each of the TG parameters in predicting significant coronary stenosis. For each AUC, a 95% CI was included as well, exploring significance abilities in predicting significant coronary stenosis.

Subgroup analyses were implemented for NSTEMI (N = 32), Unstable angina (UA; N = 15), Chest pain (N = 69), Diabetes mellitus (N = 52), HLP (N = 83), HTN (N = 63), IHD (N = 52) and overweight/obesity (N = 85). A 2-sided p-value of < .05 was considered to be statistically significant and a P-value of 0.05–0.1 was considered borderline. Statistical analysis was performed using SAS 9.4.

## Results

From April 2018 through September 2019, we recruited 128 patients with ACS or chest pain and whom were planned for coronary angiography. Ten patients did not eventually undergo angiography and were taken out of the study. Three patients did not have a calculated syntax I score due to previous coronary artery bypass surgery.

Average age was 58.9 (SD = 11.5) years old and 82% were males. Half of the patients had significant coronary stenosis in angiography, the vast majority of patients had a low syntax I score of <22 (97%). Both groups, significant vs non-significant coronary stenosis, shared many similarities in demographics and co-morbidities, yet patients with significant coronary stenosis were older and had higher rates of former IHD (Table 1).

**Table 1:** Demographics.

	Total	Significant coronary stenosis		P-value
		No	Yes	
Gender, Male	97 (82.2%)	46 (77.97%)	51 (86.44%)	0.2288
Age (years)	58.94 (11.52)	56.31 (12.37)	61.58 (10.03)	0.0123
IHD	52 (44.07%)	18 (30.51%)	34 (57.63%)	0.003
CHF	5 (4.24%)	4 (6.78%)	1 (1.69%)	0.3642
DM	52 (44.07%)	24 (40.68%)	28 (47.46%)	0.4583
Hypertension	63 (53.39%)	29 (49.15%)	34 (57.63%)	0.3562
Current or past smoker	66 (55.93%)	33 (55.93%)	33 (55.93%)	>0.99
Overweight/Obesity	85 (73.91%)	43 (74.14%)	42 (73.68%)	0.9558
Hyperlipidemia	83 (70.34%)	37 (62.71%)	46 (77.97%)	0.0697
Liver disease	0 (0%)	0 (0%)	0 (0%)	-
Lung disease	8 (6.78%)	6 (10.17%)	2 (3.39%)	0.2719
CVA/TIA	9 (7.63%)	3 (5.08%)	6 (10.17%)	0.4903
Renal failure	6 (5.08%)	4 (6.78%)	2 (3.39%)	0.6793

Mean (SD) [median, interquartile range]. IHD- ischemic heart disease, CHF-congestive heart failure, DM- diabetes mellitus, CVA- cerebrovascular accident, TIA- transient ischemic attack, NSTEMI- non-ST elevation myocardial infarction

When analyzing all 118 patients as a homogenous group, we found no statistically significant correlation between TG values and coronary stenosis, nor between TG values and the syntax I score (Tables 2 and 3). There was a positive correlation between peak height and troponin level (Spearman correlation coefficient 0.194, P-value = 0.035) (Table 4).

**Table 2:** Association between TG and significant coronary stenosis in all patients.

	Total	Significant coronary stenosis		P-value
		No	Yes	
**TG parameters**				
Lagtime (min)	4.75 (0.97) [4.67, 4–5.22]	4.67 (0.92) [4.67, 4–5.22]	4.82 (1.03) [4.67, 4.22–5.22]	0.4067
ETP (nM*min)	1936.05 (453.61) [1907.25, 1621–2215.5]	1957.46 (492.54) [1896.5, 1565.5–2237]	1914.63 (414.18) [1942.5, 1630–2209.5]	0.6101
peak height (nM)	334.17 (77.61) [332.39, 281.56–384.75]	337.74 (80.39) [326.95, 282.37–394.37]	330.59 (75.24) [341.95, 281.27–384.15]	0.6188
ttpeak (min)	7.71 (1.43) [7.39, 6.67–8.67]	7.65 (1.35) [7.22, 6.67–8.83]	7.77 (1.5) [7.44, 6.78–8.5]	0.6545

Mean (SD) [median, interquartile range]. TG- thrombin generations, Min- minutes, nM- nanomole, ETP- endogenous thrombin potential, ttpeak- time to peak

**Table 3:** Correlation between TG and syntax I score.

TG parameters	All patients N = 115		Chest pain (N = 68)		NSTEMI (N = 30)		UA (N = 15)	
	Correlation coefficient	P-value	Correlation coefficient	P-value	Correlation coefficient	P-value	Correlation coefficient	P-value
lag time	0.08	0.396	0.03	0.811	0.31	0.099	-0.11	0.692
ETP	-0.11	0.233	-0.05	0.673	-0.23	0.213	-0.34	0.219
peak height	-0.02	0.836	0.06	0.641	-0.28	0.131	-0.38	0.157
ttPeak	0.02	0.863	-0.07	0.587	0.37	0.045	0.28	0.320

Spearman correlation. TG- thrombin generations, ETP- endogenous thrombin potential, ttpeak- time to peak, UA- unstable angina, NSTEMI- non-ST elevation myocardial infarction.

**Table 4:** Correlation between TG and troponin peak.

TG parameters	All patients N = 118		Chest pain (N = 69)		NSTEMI (N = 32)		UA (N = 15)	
	Correlation coefficient	P-value	Correlation coefficient	P-value	Correlation coefficient	P-value	Correlation coefficient	P-value
lag time	0.07	0.439	0.22	0.065	0.24	0.181	0.10	0.731
ETP	0.09	0.313	0.11	0.364	0.24	0.177	0.14	0.612
peak height	0.19	0.035	0.10	0.402	0.25	0.172	0.31	0.257
Ttpeak	-0.07	0.473	0.10	0.407	0.10	0.588	-0.11	0.707

Spearman correlation. TG- thrombin generations, ETP- endogenous thrombin potential, ttpeak- time to peak, UA- unstable angina, NSTEMI- non-ST elevation myocardial infarction.

### Subgroups analysis according to diagnosis

#### Chest pain group

**Fig 1:**
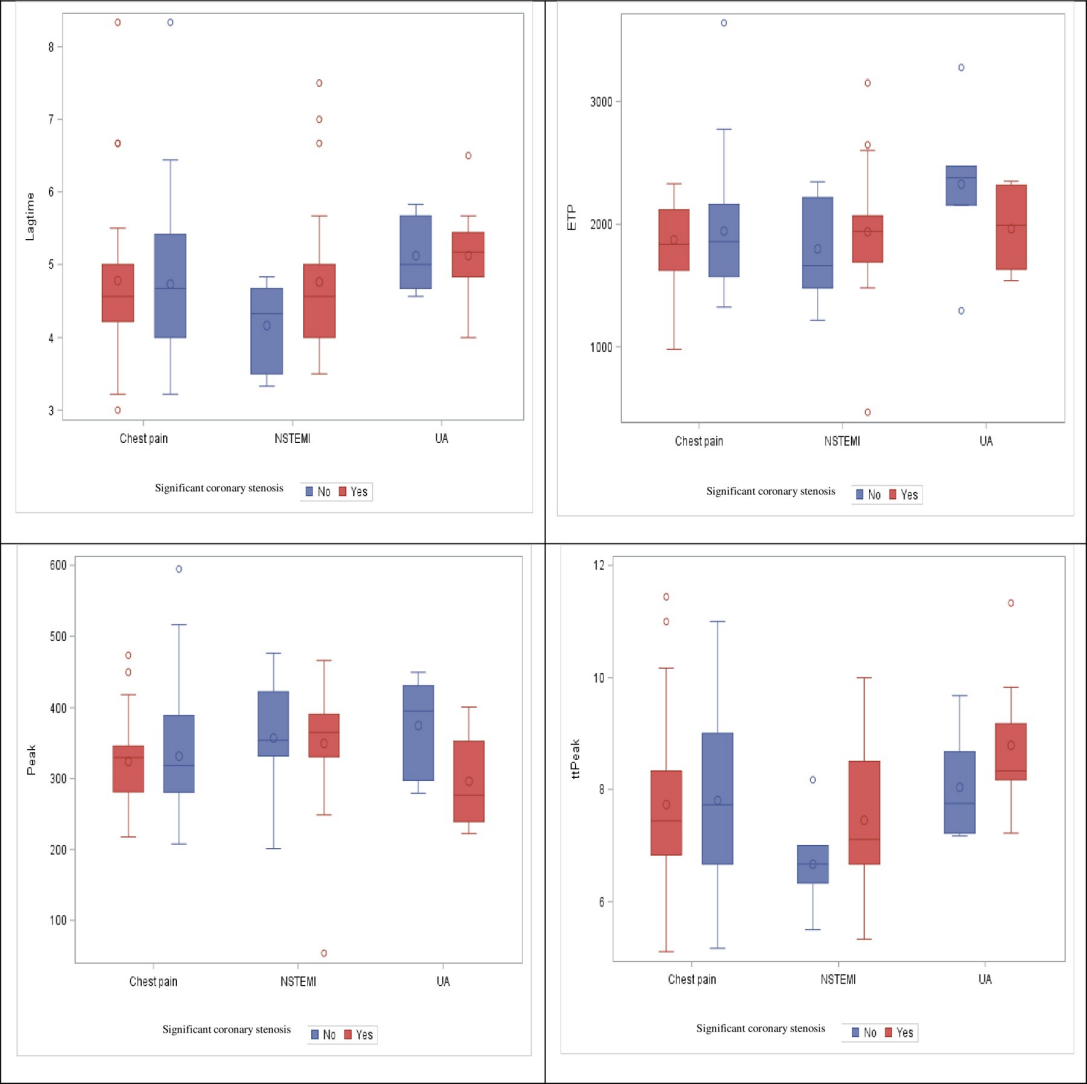
TG distribution among sub-groups, by significant coronary stenosis.

There was no significant association between TG values and significant coronary stenosis (Fig 1), nor between TG and the syntax I score (Table 3). A positive correlation between lag time and troponin (Spearman correlation coefficient 0.223, P-value = 0.065) was noticed (Table 4).

#### Unstable angina group

There was a significant difference in peak height values between patients with and without coronary stenosis (mean 296.23 [SD = 67.76] and mean 374.78 [SD = 70.59] respectively; P-value = 0.029) (Fig 1). We found no correlation between TG values and the syntax I score nor between TG and troponin (Tables 3 and 4).

#### NSTEMI group

**Fig 2:**
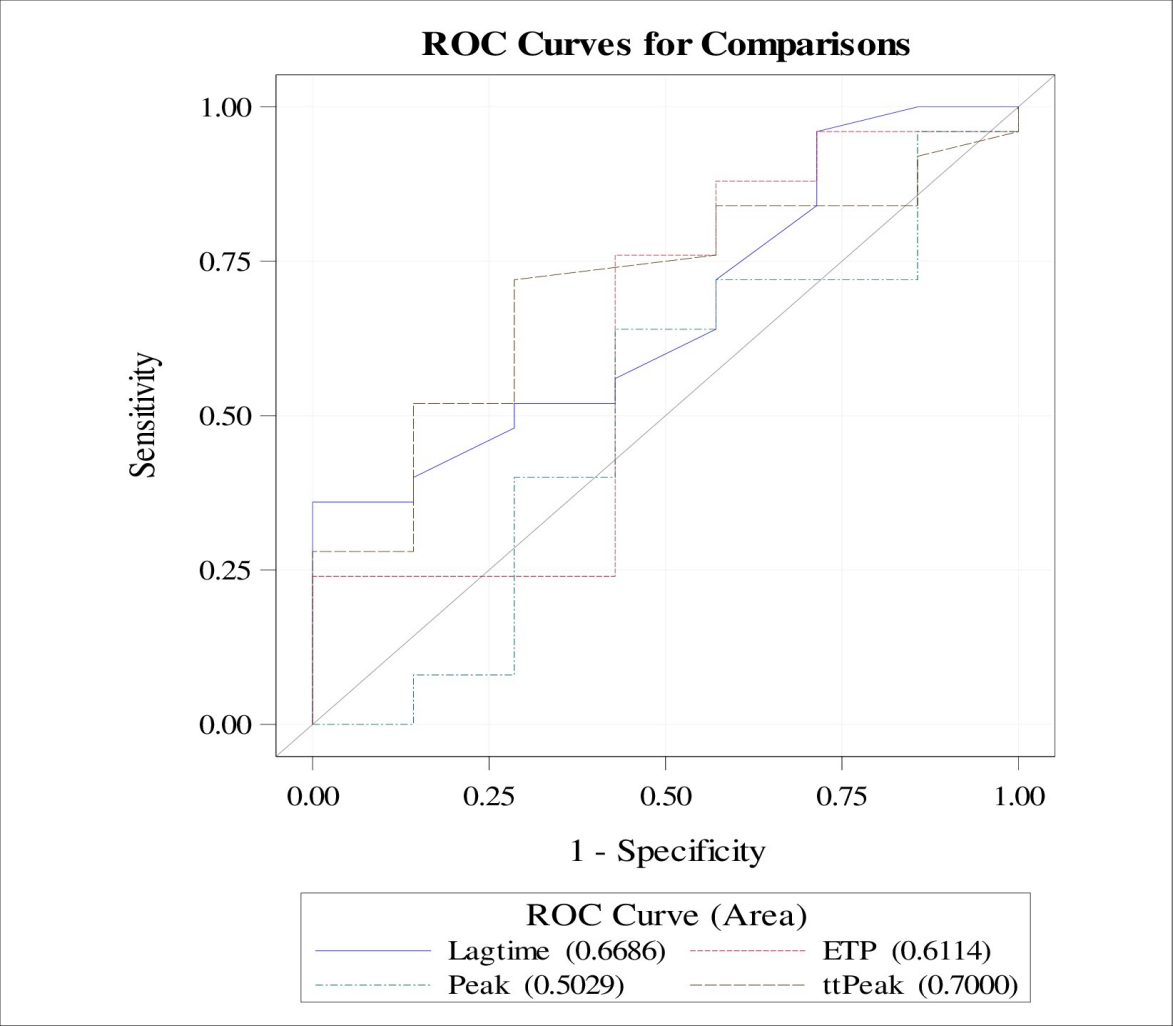
TG predictive ability of significant coronary stenosis in NSTEMI patients.

No significant associations between TG values and coronary stenosis were found (Fig 1), but the area under the ROC curve of lag time, ETP and ttpeak for significant coronary stenosis were 66.8% [95%CI: 44%-89%], 61.1% [95%CI: 33%-89%] and 70% [95%CI: 49%-91%] respectively (Fig 2), There was a positive correlation between lag time and the syntax I score and between ttpeak and the syntax I score (Spearman correlation coefficient 0.31, P-value = 0.099 and Spearman correlation coefficient 0.37, P-value = 0.045 respectively) (Table 3). There was no significant correlation between TG values and troponin in this subgroup (Table 4).

### Subgroups analysis according to co-morbidities

In patients with former IHD (N = 52), there was a borderline difference in ETP values between those with and without coronary stenosis (mean 1867.3 [SD = 334.9] and mean 2160.1 [SD = 641.7] respectively; P-value = 0.084), and a borderline negative correlation between ETP and the syntax I score (Spearman correlation coefficient -0.23, P-value = 0.113).

The average follow-up time was 9.3 months. Fourteen patients (12%) had MACE during follow-up. There was no difference in TG values according to MACE.

## Discussion

Our results showed that when analyzing the entire study population, there is no association between TG values and coronary stenosis severity. In sub-group analysis, it seems that in patients with chest pain (without ACS diagnosis), there is no association between TG values and coronary stenosis severity. In patients with ACS, several TG values were associated with coronary stenosis, however this trend did not repeat itself consistently. In patients with former IHD, we found a non-conclusive association between TG and coronary stenosis severity. We found a correlation between peak height and troponin levels in the entirety of the patient’s population. Seemingly, the greater severity of myocardial injury may correlate with a greater ability to generate thrombin. However, these results were not repeated in all TG values, causing this conclusion to remain uncertain.

Past studies showed that in-vivo thrombin activity can correlate with the presence of coronary artery disease, with disease severity assessed using angiogram or computed tomography (CT), and with mortality in IHD patients [5]. Studies evaluating thrombin activity ex-vivo showed less conclusive results. Some studies showed increased TG activity in ACS patients, however, the correlation between TG and disease severity was not studied [6,7]. Other studies did not find a correlation between TG and incidence of IHD, yet these studies included only elderly patients and evaluated only ETP and peak height CAT values [8,9]. Julian et al showed that although thrombin tests in-vivo can predict coronary atherosclerosis by CT angiogram, ex-vivo tests (ETP) did not predict coronary disease. However, this study did not include other CAT values [2].

As opposed to past studies, our current study is unique and innovative as it uses all CAT values for evaluating thrombotic phenotype and is the first to use coronary angiogram, the “gold standard” technique, to evaluate the association between TG and IHD. The need to find a new, easily accessible, non-invasive tool, such as the CAT assay, for prediction of coronary stenosis seems highly important and compelling. Our results show that CAT does not correlate well with coronary stenosis in patients with chest pain but may correlate and be of value in ACS patients. Theoretically, it seems reasonable that in patients with chest pain and no signs of acute myocardial injury, there will be no activation of the coagulation system and, therefore, no disrupted TG values, while in patients with ACS there will be an activation of the coagulation system and, therefore, a change in TG formation ability corresponding with disease severity. Essentially, in this study, we learned that there is no convincing association between TG and coronary stenosis, evaluated by coronary angiogram. This is important as our results strengthen the former understanding that, in contrast to in-vivo tests, ex-vivo TG tests examine the potential capacity to form thrombin rather than evaluating a formed thrombotic state [3]. Perhaps, an acute thrombus formation in the coronary arteries only constitutes a local process, not effecting the thrombotic phenotype systemically, and therefore, not reflected in the TG values that were withdrawn from a peripheral vein, as were in our study. Yet, due to the curious correlation found in ACS sub-groups, and the beneficial potential in finding a new non-invasive tool for an initial screening for patients with suspected coronary stenosis, we encourage further research based on the protocol and evidence gathered here.

As for TG prognostic ability, we found no correlation between TG values and the presence of MACE, therefore, it seems ex-vivo TG tests have no prognostic prediction ability. That being said, results should be interpreted with caution due to the low number of MACE in this study. Past research by Attanasio et al discovered that there is a positive correlation between ETP and peak height, and mortality in ACS patients [1]. The different results may be explained by our study’s patient composition, which was more diverse, including ACS as well as chest pain patients (without evidence of myocardial injury).

Limitation: Study population was a mixture of ACS and non-ACS patients. Only 12% diagnosed with UA and 27% with NSTEMI. This combination might impair the ability to find a statistically significant correlation between TG and severity of coronary disease in the whole study population. Moreover, the study did not include STEMI patients. Another limitation was the use of a PPP for TG testing, thereby excluding platelets effect.

## Conclusion

TG values do not predict coronary stenosis severity in patients without evidence of myocardial injury, but may have prediction abilities in patients with established IHD and ACS. Furthermore, TG may correlate with troponin peak level. The potential of CAT as a non-invasive tool for the initial screening for suspected coronary stenosis in specific sub-populations should be further studied.
